# Effect of Statins on the Progression of Coronary Calcification in Kidney Transplant Recipients

**DOI:** 10.1371/journal.pone.0151797

**Published:** 2016-04-21

**Authors:** Daniel Constantino Yazbek, Aluizio Barbosa de Carvalho, Cinara Sá Barros, Jose Osmar Medina Pestana, Maria Eugênia F. Canziani

**Affiliations:** Nephrology Division, Federal University of São Paulo, São Paulo, SP, Brazil; University of Milan, ITALY

## Abstract

**Background:**

Coronary calcification (CAC) is highly prevalent in kidney transplant recipients (KTRs) and has been associated with cardiovascular morbidity and mortality. Some studies have shown a reduction in CAC progression with statin therapy in the general and chronic kidney disease (CKD) populations.

**Objectives and Methods:**

The aim of the present study was to evaluate the effect of statins on CAC progression in incident kidney transplant recipients. Patients were randomly assigned to the statin (n = 61, 10 mg daily) and control group (n = 59). CAC and biochemical analyses were performed at baseline and 12 months.

**Results:**

At baseline, CAC was observed in 30% and 21% of patients in the statin and control groups, respectively (p = 0.39). The calcium score at baseline and its absolute and relative changes over 12 months of follow up were similar among the groups. In the statin group, total cholesterol (p < 0.001), low density lipoprotein cholesterol (p < 0.001) and triglycerides (p = 0.005) decreased, and the estimated glomerular function rate increased (p<0.001) significantly. CRP levels remained stable (p = 0.52) in the statin group but increased in the control group (p = 0.01). In the multivariate model, there was no difference in CAC progression between the groups (group effect p = 0.034; time-effect p = 0.23; interaction p = 0.74). Similar results were obtained when only patients with ≥ 10AU calcium score (calcified) were analyzed (group effect p = 0.051; time-effect p = 0.58; interaction p = 0.99).

**Conclusion:**

Although statins reduce the levels of cholesterol, triglycerides, inflammation and improve graft function, the dose adopted in the current study did not delay CAC progression within 12 months of follow up.

**Trial Registration:**

Brazilian Clinical Trials Registry RBR-32RFMB

## Introduction

Kidney transplantation is the preeminent treatment for end-stage renal disease patients. It has been suggested that kidney transplant recipients (KTRs) have less comorbidities, improved quality of life and better survival compared to patients receiving long-term dialysis therapy [1–3]. Thus, a successful transplantation could be presumed to decrease the occurrence and progression of uremia-related complications by restoring renal function. However, KTRs have a 4-fold greater risk of cardiovascular disease (CVD) than the general population [4]. Mortality among this population has been shown to be substantially high, particularly in the first month after transplantation, and CVD is the absolute leading cause of death [1, 5, 6]. Coronary artery calcification (CAC) constitutes an important marker of vascular damage that is highly prevalent among KTRs [7] and has a strong relationship with cardiovascular events and death [8].

Two important trials using statins in patients receiving hemodialysis (4D and AURORA), [9,10] and one trial in patients with renal transplant (ALERT) [11] failed to demonstrated a benefit in primary cardiovascular outcomes. Recently, the Study of Heart and Renal Protection (SHARP) showed that lowering low-density lipoprotein cholesterol (LDL-c) by using a combination of simvastatin and ezetimibe safely reduced the risk of atherosclerotic events in a wide range of patients with chronic Kidney disease (CKD) [12]. In addition, a recent post hoc analysis of the AURORA study demonstrated that rosuvastatin significantly reduced the rate of cardiac events (32%) among diabetic patients undergoing hemodialysis [13]. Current guidelines for treating dyslipidemia in CKD suggest treatment with a statin to prevent coronary disease in this population [14].

Of note vascular calcification involves multiple complex pathways in CKD patients, although it is expected that successful kidney transplantation will improve uremia and mineral bone metabolism, dyslipidemia and inflammation are frequently observed in this population and can contribute to CVD. Therefore statins could be one of the therapeutic approaches in the prevention of CAC progression. Few interventional studies on the development of CVD enrolled KTRs, and to the best of our knowledge, there is no clinical trial to date evaluating the association of statins with CAC progression in this population. Thus, the present study aimed to evaluate the impact of statins on the progression of coronary calcification in incident kidney transplant recipients.

## Subjects and Methods

### Subjects

One hundred and twenty incident KTRs were recruited from the outpatient Transplant Unit of the Federal University of São Paulo (Brazil) in the period 26/09/2007 at 23/06/2009 in a follow up of one year by June 2010. Patients who had underwent kidney transplantation within 60 days were approached to participate in the study. The exclusion criteria were age less than 18 years or greater than 60 years, creatinine clearance less than 30 ml/min, patients prioritized for kidney transplantation and patients who had experienced any cardiovascular event or received statin 3 months prior to transplantation.

According to the local protocol, all of the patients underwent initial immunosuppression with prednisone. Seventy-nine patients (66%) were KTRs of living donors (HLA I 22%, HLA II 35%, and HLA III 8%). KTRs of living donors with human leukocyte antigen I (HLA—totally matched) received cyclosporine and azathioprine, HLA II (partially matched) and III (fully mismatched) used both tacrolimus and azathioprine. Of note, only one preemptive transplantation was performed. The KTRs of deceased donors were induced with basiliximab and received tacrolimus and mycophenolic acid. Thymoglobulin was used when patients had a higher panel reactive antibodies (> 20%). No patients were using vitamin K antagonist during the study.

The study was reviewed and approved in 02/03/2007 by the Ethics Advisory Committee of the Federal University of São Paulo, and each patient signed the informed consent form. Important to note, no deviation occurred in the duration of the study protocol. This study were registred at The Brasilian Clinical Trials Registry (REBEC—www.ensaiosclinicos.gov.br/) after the beginning the study, under the RBR-32RFMB number. The delay in registration was because at the time of the beginning of the study there was no available the REBEC platform. In addition, the authors confirm that all ongoing and related trials for this drug/intervention are registered.

### Study protocol

In this open-label prospective study of 12-month the patients were selected and randomized to receive statin or to compose the control group. An independent researcher generated a computerized random list and the allocation sequence was concealed in closed box of 20 patients. At baseline and after 12 months, blood tests and a cardiovascular assessment were performed within a one month interval.

Sixty-one patients received a statin, 45 of them received 10 mg/day of rosuvastatin (Astra Zeneca AB, Södertälje, Sweden). Due to a logistic problem in the rosuvastatin supply, 16 patients (the last ones included in the study) received 10 mg/day of atorvastatin (Pfizer, New York, USA) [15]. No difference in the baseline demographics or clinical and laboratory characteristics were found between patients receiving rosuvastatin compared to those taking atorvastatin. The patients maintained the same statin regimen during the entire follow-up period. The LDL cholesterol was considered on therapeutics goals when was under 100 mg/dl.

Previous CVD was defined as a documented medical history of cerebrovascular, peripheral arterial or venous disease, myocardial infarction, angina pectoris, coronary artery revascularization, or a positive result on the diagnostic test (ultrasonography, stress test, coronary angiography or radionuclide imaging). Arterial pressure was measured in all patients, and hypertension was defined as a systolic blood pressure > 130 mmHg and/or diastolic blood pressure > 80 mmHg or the use of antihypertensive drugs. Body mass index (BMI) was calculated as body weight divided by squared height. Waist circumference was assessed at the umbilicus level, using the mean of 3 measurements.

### Cardiovascular disease assessment

The patients underwent coronary artery calcification (CAC) examination by a multi-slice computed tomography scanner (LightSpeed^®^ Pro16 –GE Healthcare, Milwaukee, USA) using a gantry rotation of 0.4 s, collimation of 2.5 mm (slice thickness), and reconstruction time of 6 frames per second. A calcium threshold of ≥130 Hounsfield units (HU) was used. The images were scored by a single radiologist blinded to all clinical and biochemical aspects of the patient. As described by Agatston, the calcium score was determined by multiplying the area of each calcified lesion by a weighing factor corresponding to the peak pixel intensity for each lesion [16]. The sum of each lesion in all of the coronary arteries was used for analysis. The presence of CAC was defined as a calcium score ≥10 AU and severe CAC as a calcium score ≥400 AU. The absolute increase in the calcium score indicated the difference between the 12-month and baseline values. The absolute increase in the delta calcium score was calculated using the 12-month score minus the baseline value. The relative increase in the delta calcium score was calculated as the ratio of the absolute increase to the baseline value multiplied by 100. CAC progression was defined as a delta CAC greater than zero.

Two-dimensional color Doppler echocardiography (Philips^®^ HDI 5000, Royal Philips Electronics, Netherlands) was performed according to the recommendations of the American Society of Echocardiography [17]. Left ventricular hypertrophy (LVH) was considered to be present for a left ventricular mass index > 115 g/m^2^ among men and > 95 g/m^2^ among women. Systolic dysfunction was defined as an ejection fraction ≤ 55%.

### Laboratory data

Blood samples were drawn in the fasting state to determine the following laboratory tests: blood count, serum creatinine, glucose, lipid profile, ionized calcium (reference: 1.20 to 1.40 mmol/L), serum phosphorus (reference: 3.5 to 4.5 mg/dl), total alkaline phosphatase (reference: 40 to 129 U/L for males, 35 to 104 U/L for females), intact parathyroid hormone (iPTH—Immulite Assay, DPC, Los Angeles, CA, USA; reference 10 to 65 pg/mL), pH, bicarbonate and C-reactive protein (CRP, immunometric assay, Immulite^®^). Proteinuria and phosphaturia were measured on obtaining 24 h urine samples. Abnormal proteinuria was defined as a urinary protein excretion > 0.15 g/24 h. Creatinine clearance was estimated by the CKD-EPI equation [18].

### Statistical analysis

The mean and standard deviation, median and interquartile range or frequency (proportion) were calculated for the variables, as appropriate. The Kolmogorov-Smirnov statistical test was used to investigate the normal distribution of data. Comparisons of continuous variables between groups were performed using Student’s *t*-test and the Mann-Whitney U-test, and within groups using Student’s *t*-test or Wilcoxon, for normal and skewed data, respectively. Comparisons of proportions were performed using chi-squared analysis or Fischer’s exact test, as appropriate. A Generalized Estimation Equation (GEE) was performed to identify the variables associated with calcification progression. The multivariate model was adjusted for male gender, body mass index, prior dialysis time and lipid profile. A Tweedie distribution was assumed in the model because the calcium score had an asymmetric distribution and many values with zeros. *P* values < 0.05 were considered to be statistically significant. All of the statistical analyses were performed using SPSS for Windows version 15.0 (SPSS Inc, Chicago, IL). Based on Seyahi *et al* [19], a sample size of 57 participants per group was estimated using Gpower V 3.1 software, to provide a two-sided significance of 5% with 80% power to detect 50% difference in coronary calcium score between the group after one year. Considering 6% of losses in one year, we aimed to randomize 120 subjects.

## Results

**Fig 1:**
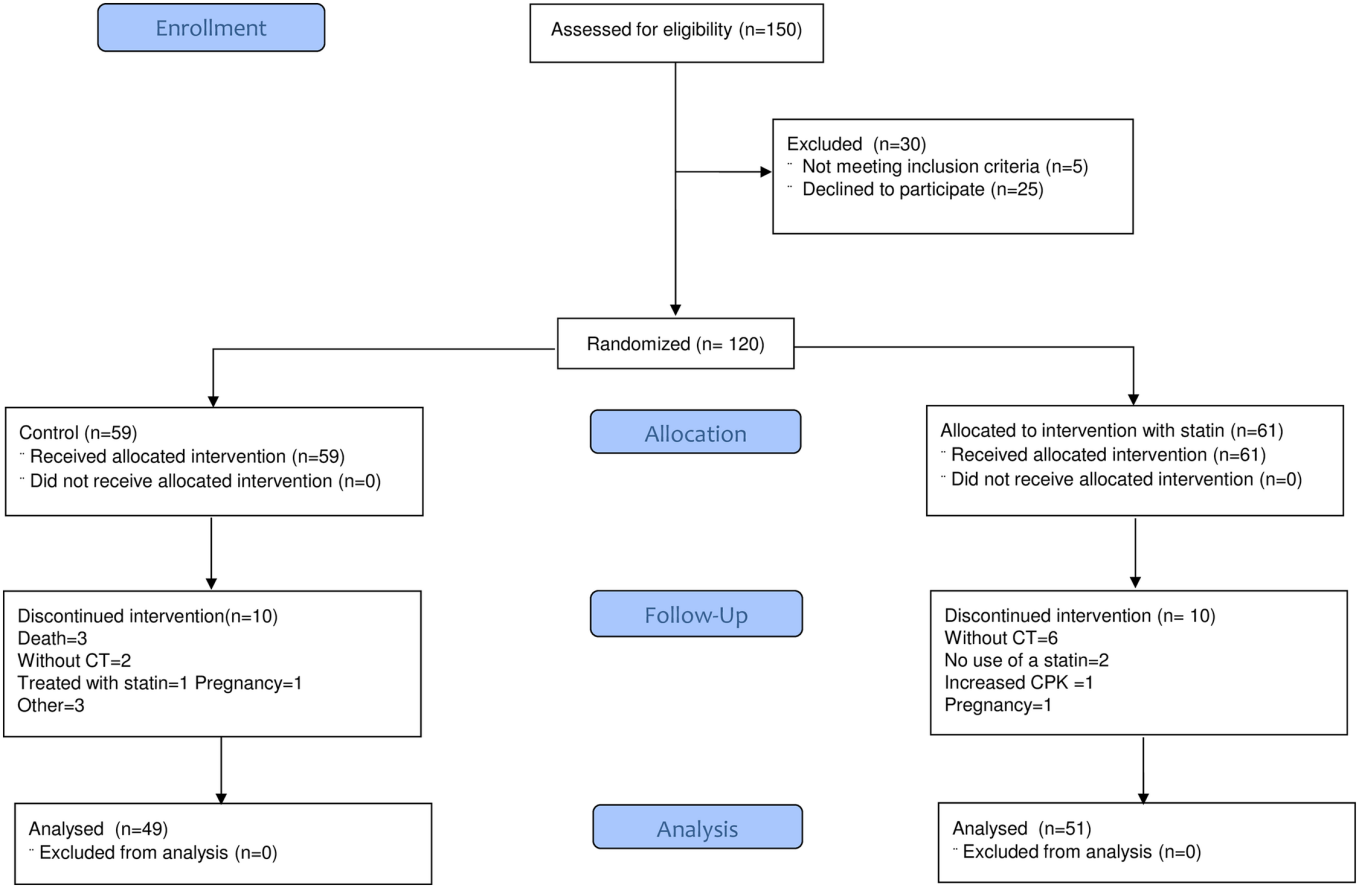
Patient distribution.

The patient distribution by treatment group is shown in Fig 1. Twenty patients did not complete the study (10 in each group). Only one patient discontinued use of statin due to increase in CPK levels. The clinical and laboratory characteristics of the remaining patients and those who withdrew were similar, except for age (41.1 ± 10.0 vs. 36.0 ± 8.8 years p = 0.04, respectively). One hundred patients completed the study protocol, with two available multislice coronary tomography.

Table 1 depicts demographic and laboratory characteristics at baseline and the 12-month follow up in the patients who completed the study. In the control group, comparing the baseline and 12-month data, the patients had an increased BMI, waist circumference, hemoglobin, phosphate, pH, bicarbonate, and C-reactive protein and decreased levels of tacrolimus, cyclosporine, total and HDL cholesterol, ionized calcium, alkaline phosphatase and left ventricular index. There was also a trend to increase phosphaturia in these patients.

**Table 1:** Comparison of demographic and laboratory parameters in the statin and control groups.

	Control (n = 49)			P	Statin (n = 51)			P	Between groups	
	Baseline	12 months	Delta		Baseline	12 months	Delta		P^T0^	P^T12^
**Age (years)**	41.0 ± 9.7				41.2 ± 10.5					
**Male n (%)**	32 (65)				24 (47)				**0.07**	
**Smoking n (%)**	3 (6)				5 (10)				**0.71**	
**Hypertension n (%)**	45 (92)				44 (86)				**0.37**	
**Diabetes n (%)**	5 (10)				2 (4)				**0.26**	
**Sedentary n (%)**	40 (78)				39 (80)				**0.89**	
**Current medication n (%)**										
**ACE- inhibitors/ARB**		14 (29)				15 (29)				**0.47**
**β-blockers**		21 (43)				26 (51)				**0.27**
**Oral antidiabetic**		5 (10)				8 (16)				**0.30**
**Insulin**		9 (18)				2 (4)				**0.02**
**Time of renal transplant**	45.7 ± 11.7				42.1 ± 9.5				**0.07**	
**Prior dialysis time (months)**	18 (9–38)				28 (14–66)				**0.03**	
**KTRs with deceased donors n (%)**	12 (24)				21 (41)				**0.08**	
**BMI (kg/m**^**2**^**)**	22.9 ± 3.5	25.3 ± 4.2	+2.4	**<0.001**	24.8 ± 4.5	26.9 ± 5.2	+2.1	**<0.001**	**0.02**	**0.10**
**WC (cm)**	86.6 ± 10.0	91.7 ± 10.5	+5.1	**<0.001**	88.7 ± 11.5	94.1 ± 13.2	+5.4	**<0.001**	**0.32**	**0.32**
**SBP (mmHg)**	132.2 ± 14.3	130.2 ± 15.3	-2.0	**0.49**	131.2 ± 15.2	129.4 ± 12.7	**-1.8**	**0.46**	**0.72**	**0.78**
**DBP (mmHg)**	85.1 ± 10.2	83.3 ± 9.0	-1.8	**0.34**	82.3 ± 10.3	82.0 ± 8.7	**-0.3**	**0.83**	**0.18**	**0.46**
**Hemoglobin (mg/dl)**	13.4 ± 1.9	14.3 ± 1.7	+0.9	**0.009**	12.5 ± 1.8	13.9 ± 1.8	**+1.4**	**<0.001**	**0.02**	**0.32**
**Creatinine (mg/dl)**	1.30 ± 0.28	1.25 ± 0.30	-0.05	**0.19**	1.41 ± 0.48	1.24 ± 0.46	**-0.17**	**<0.001**	**0.14**	**0.86**
**CKD EPI (ml/min/1.73 m**^**2**^**)**	66.7 ± 19.5	69.8 ± 18.7	+3.1	**0.13**	59.2 ± 19.0	69.3 ± 21.0	**+10.1**	**<0.001**	**0.053**	**0.90**
**Tacrolimus (mg/dl)**	10,1 ± 4,4	6,8 ± 2,1	-3.3	**<0.001**	10,3 ± 3,6	6,8 ± 2,6	**-3.5**	**<0.001**	**0.87**	**0.97**
**Ciclosporine (mg/dl)**	115 ± 52	85 ± 38	-30	**<0.001**	115 ± 54	76 ± 37	**-39**	**0.08**	**0.99**	**0.57**
**Glucose (mg/dl)**	88 (79–98)	87 (82–98)	-1	**0.45**	89 (80–97)	92 (84–99)	**+3**	**0.11**	**0.87**	**0.31**
**Total cholesterol (mg/dl)**	189.2 ± 33.2	173.6 ± 31.9	-15.6	**0.002**	215.0 ± 46.1	160.8 ± 41.7	**-54.2**	**<0.001**	**0.002**	**0.09**
**HDL-c (mg/dl)**	59.9 ± 15.7	46.6 ± 14.7	-13.3	**<0.001**	53.1 ± 16.1	45.3 ± 13.3	**-7.8**	**<0.001**	**0.07**	**0.65**
**LDL-c (mg/dl)**	102.2 ± 26.7	99.3 ± 24.9	-2.9	**0.40**	118.9 ± 35.3	87.2 ± 37.8	**-31.7**	**<0.001**	**0.01**	**0.06**
**Triglycerides (mg/dl)**	131 (100–173)	123 (84–174)	-8	**0.93**	179 (133–232)	157 (90–209)	**-22**	**0.005**	**0.001**	**0.17**
**Ionized Ca (mmol/l)**	1.40 (1.34–1.44)	1.35 (1.31–1.40)	-0.05	**<0.001**	1.40 (1.33–1.45)	1.36 (1.32–1.40)	**-0.04**	**0.01**	**0.99**	**0.35**
**Phosphate (mg/dl)**	2.6 ± 0.6	3.2 ± 0.6	+0.6	**<0.001**	2.8 ± 0.9	3.3 ± 0.7	**+0.5**	**0.001**	**0.25**	**0.62**
**AP (U/l)**	96 (75–133)	81 (59–104)	-5	**0.001**	91 (75–185)	80 (58–108)	**-11**	**<0.001**	**0.67**	**0.73**
**iPTH (pg/ml)**	63 (36–93)	71 (40–1137)	+8	**0.14**	67 (40–129)	71 (45–113)	**+4**	**0.95**	**0.47**	**0.65**
**pH**	7.30 ± 0.05	7.33 ± 0.05	+0.03	**0.003**	7.30 ± 0.06	7.33 ± 0.04	**+0.03**	**0.004**	**0.89**	**0.97**
**Bicarbonate (mmol/l)**	26 (23–28)	27 (25–29)	+1	**0.009**	24 (21–27)	26 (24–28)	**+2**	**0.003**	**0.16**	**0.20**
**C-reactive protein (mg/l)**	0.08 (0.03–0.27)	0.19 (0.09–0.48)	+0.11	**0.01**	0.09 (0.04–0.36)	0.19 (0.09–0.53)	**+0.10**	**0.52**	**0.30**	**0.78**
**Proteinuria (mg/24 h)**	0 (0–0.19)	0 (0–0)	0	**0.78**	0 (0–0)	0 (0–0.04)	**0**	**0.72**	**0.79**	**0.83**
**Phosphaturia (mg/24 h)**	635 (493–775)	667 (518–908)	+32	**0.07**	553 (416–809)	615 (455–813)	**+62**	**0.98**	**0.51**	**0.20**
**Left atrium (mm)**	37.4 ± 5.3	36.7 ± 4.7	-0.7	**0.73**	38.7 ± 5.1	37.6 ± 4.8	**-1.1**	**0.26**	**0.29**	**0.44**
**Ejection fraction**	0.67 (0.63–0.71)	0.67 (0.63–0.73)	0	**0.50**	0.68 (0.64–0.71)	0.68 (0.65–0.74)	**0**	**0.95**	**0.81**	**0.57**
**LV index (g/m**^**2**^**)**	116 (93–155)	97 (74–124)	-19	**0.01**	123 (100–159)	105 (79–116)	**-18**	**<0.001**	**0.52**	**0.49**

Mean ± standard deviation, median (interquartiles)

ACE—Angiotensin-converting enzyme; ARB—Angiotensin receptor blockers; KTR—kidney transplant recipients; BMI—body mass index; WC—waist circumference; SBP—systolic blood pressure; DBP—diastolic blood pressure; HDL-c—HDL cholesterol; LDL-c—LDL cholesterol; Ca—calcium; AP—alkaline phosphatase; iPTH—intact parathyroid hormone; CRP—C-reactive protein; LV—left ventricular; CAC—coronary calcification.

In the statin group, comparing the baseline and 12-month data, the patients had an increased BMI, waist circumference, hemoglobin, estimated glomerular function, phosphate, pH, bicarbonate and decreased creatinine, levels of tacrolimus, cyclosporine, total, LDL and HDL cholesterol, triglycerides, ionized calcium, alkaline phosphatase and left ventricular index.

**Fig 2:**
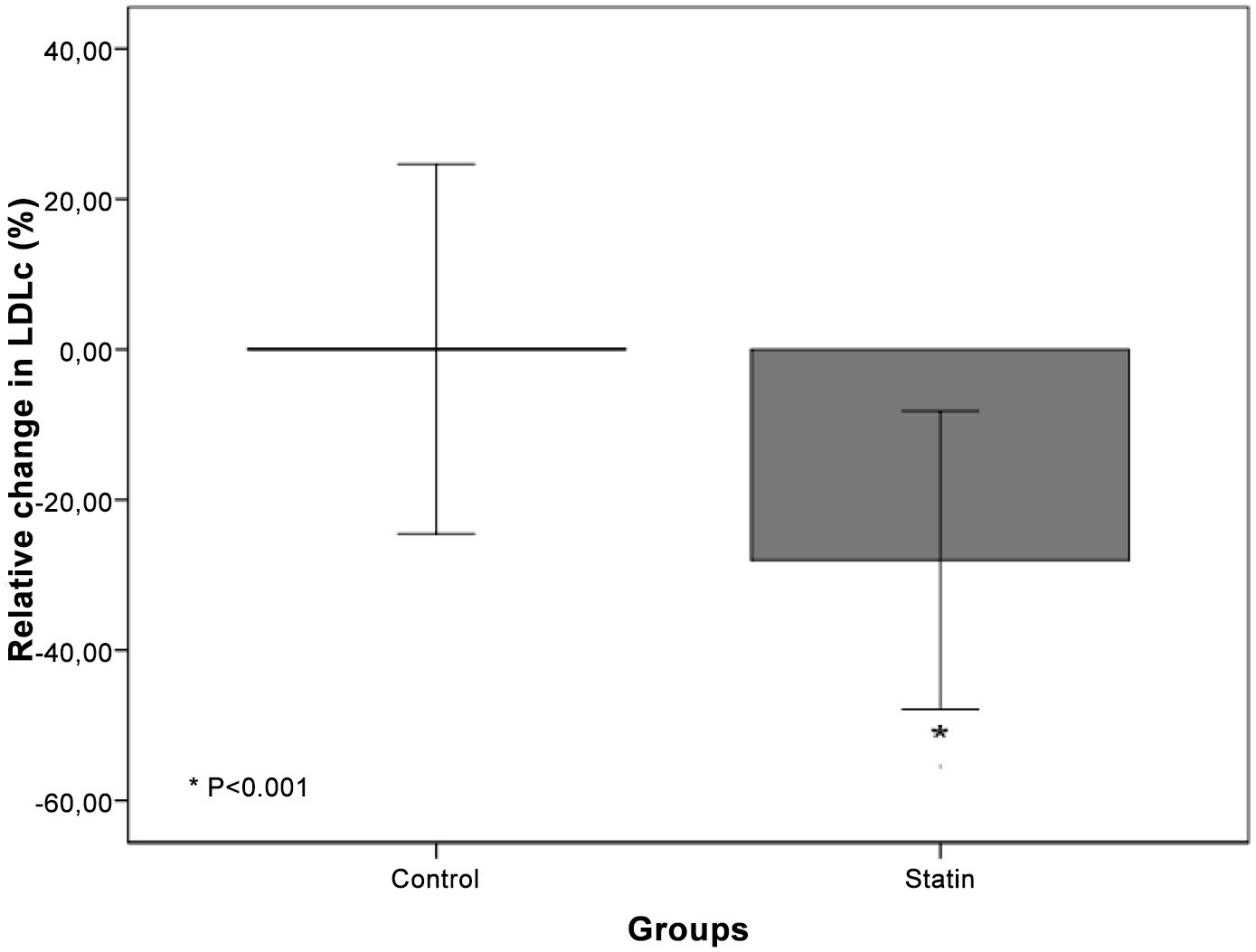
Relative Changes in LDL cholesterol.

Comparing the groups at baseline, the statin group had a longer duration of previous dialysis, increased BMI, total and LDL cholesterol, triglycerides and decreased hemoglobin. There was a trend toward a higher prevalence of KTRs with deceased donors, lower prevalence of male gender, and decrease in the estimated glomerular function rate by the CKD-EPI formula and HDL cholesterol in the statin group. There was no difference of end stage renal disease cause between the two groups. After 12 months, the statin group had a trend toward decreased total and LDL cholesterol. The recommended level of LDL cholesterol were achieved in 37 (61%) patients in the statin and 24 (39%) in control group (p = 0.01). Fig 2 depicted the relative change of LDL cholesterol during the study in both groups. There was a significant decrease of relative changes of LDL cholesterol in statin group while no change was observed in control group [-27.6% (-45.3–12.9) vs -2.1% (-18.4 14.8), p<0.001].

No difference in the baseline demographics or clinical and laboratory characteristics were found between patients receiving rosuvastatin compared to those taking atorvastatin (Table 2). Of note, there were no difference, in relative change of LDL cholesterol between the subgroups rosuvastatin and atorvastatin [-28.1% (-47.2–9.4) vs. -27.4% (-37.4–19.0), p = 0,96].

**Table 2:** Comparison on rosuvastatin and atorvastatin beteween the groups.

	Atorvastatin		Rosuvastatin		P^T0^	P^T12^
	Baseline (n = 16)	12 months (n = 16)	Baseline (n = 43)	12 months (n = 39)		
**Creatinine (mg/dl)**	1.31 ± 0.47	1.19 ± 0.29	1.41 ± 0.46	1.26 ± 0.50	**0.44**	**0.62**
**CKD EPI (ml/min/1.73 m**^**2**^**)**	64.9 ± 22.6	68.7 ± 15.0	61.0 ± 20.6	70.2 ± 23.1	**0.52**	**0.82**
**Total cholesterol (mg/dl)**	203.9 ± 43.1	153.6 ± 41.1	216.2 ± 45.8	44.6 ± 13.9	**0.35**	**0.45**
**HDL-c (mg/dl)**	51.6 ± 15.4	46.8 ± 10.2	53.5 ± 16.8	44.6 ± 13.9	**0.69**	**0.56**
**LDL-c (mg/dl)**	110.8 ± 31.3	78.2 ± 35.6	119.6 ± 35.7	90.8 ± 39.6	**0.40**	**0.28**
**Triglycerides (mg/dl)**	187 (130–234)	127 (91–184)	179 (124–246)	164 (90–213)	**0.98**	**0.33**
**CAC score (AU)**	0 (0–8.7)	0 (0–20)	0 (0–74)	0 (0–79.7)	**0.19**	**0.43**
**ΔCAC relative**		0 (0–48)		0 (0–23)		**0.49**

Mean ± standard deviation, median (interquartiles)

HDL-c—HDL cholesterol; LDL-c—LDL cholesterol; CAC—coronary calcification.

CAC score ≥ 10AU (presence of coronary calcification) at baseline was observed in 32 patients (32%), of whom 10 (10%) had severe calcification (CAC score ≥ 400AU). In the control group, 13% had CAC score ≥ 10 AU (coronary calcification) at baseline, and 19% in the statin group did (p = 0.39). At baseline, severe calcification (CAC score ≥ 400AU) was found in 6% and 4% in the control and statin groups, respectively (p = 0.74). A comparison of coronary calcification is shown in Table 3. There was a trend toward increased coronary calcification only in the control group (p = 0.06). When only the patients with CAC score ≥ 10AU (presence of coronary calcification) at baseline were considered, 20 out of 36 (62.5%) had CAC progression, whereas 63% in the statin and 61% in the control groups had progression (p = 1.0). No difference in CAC values were observed between the statin and control groups. Of note, no difference in CAC progression was observed comparing the patients receiving rosuvastatin or atorvastatin (p = 0.99). Interestingly among patient without CAC at baseline no one developed calcification during the followup.

**Table 3:** Comparison of coronary calcification (CAC score—AU) in the statin and control groups, considering calcified (≥ 10 AU), non-calcified and all patients.

	Control				Statin				Between groups	
	Baseline	12 months	Delta	P	Baseline	12 months	Delta	P	P^T0^	P^T12^
Calcified patients*	228 (70–900)	248 (105–1200)	13.0 (-5–31)	0.10	182 (18–290)	181 (57–330)	24 (-12–66)	0.25	0.42	0.23
	562.9 ± 718.6	647.0 ± 727.8	15.1 ± 27.8		358 ± 662.4	390.4 ± 763.9	59.0 ± 119.4			
Non-Calcified patients**	0 (0–3)	0 (0–7)	0 (0–4)	0.02	0 (0–24)	0 (0–8)	0 (-16–0)	0.16	0.50	0.87
	0.1 ± 0.5	0.5 ± 1.5	0.4 ± 1.0		1.0 ± 4.4	0.6 ± 1.6	-0.4 ± 1.8			
**All patients**	0 (0–77)	0 (0–123)	0 (0–0)	0.06	0 (0–74)	1 (0–96)	0 (0–23)	0.14	0.29	0.44
	183.9 ± 482.1	211.6 ± 509.3	19.6 ± 74.0		148.1 ± 455.0	161.1 ± 520.5	24.8 ± 85.4			

*N = 33 patients (13 in control and 20 in statin group).

** N = 67 patients(36 in control and 31 in statin group).

Mean ± standard deviation, median (interquartile).

**Fig 3:**
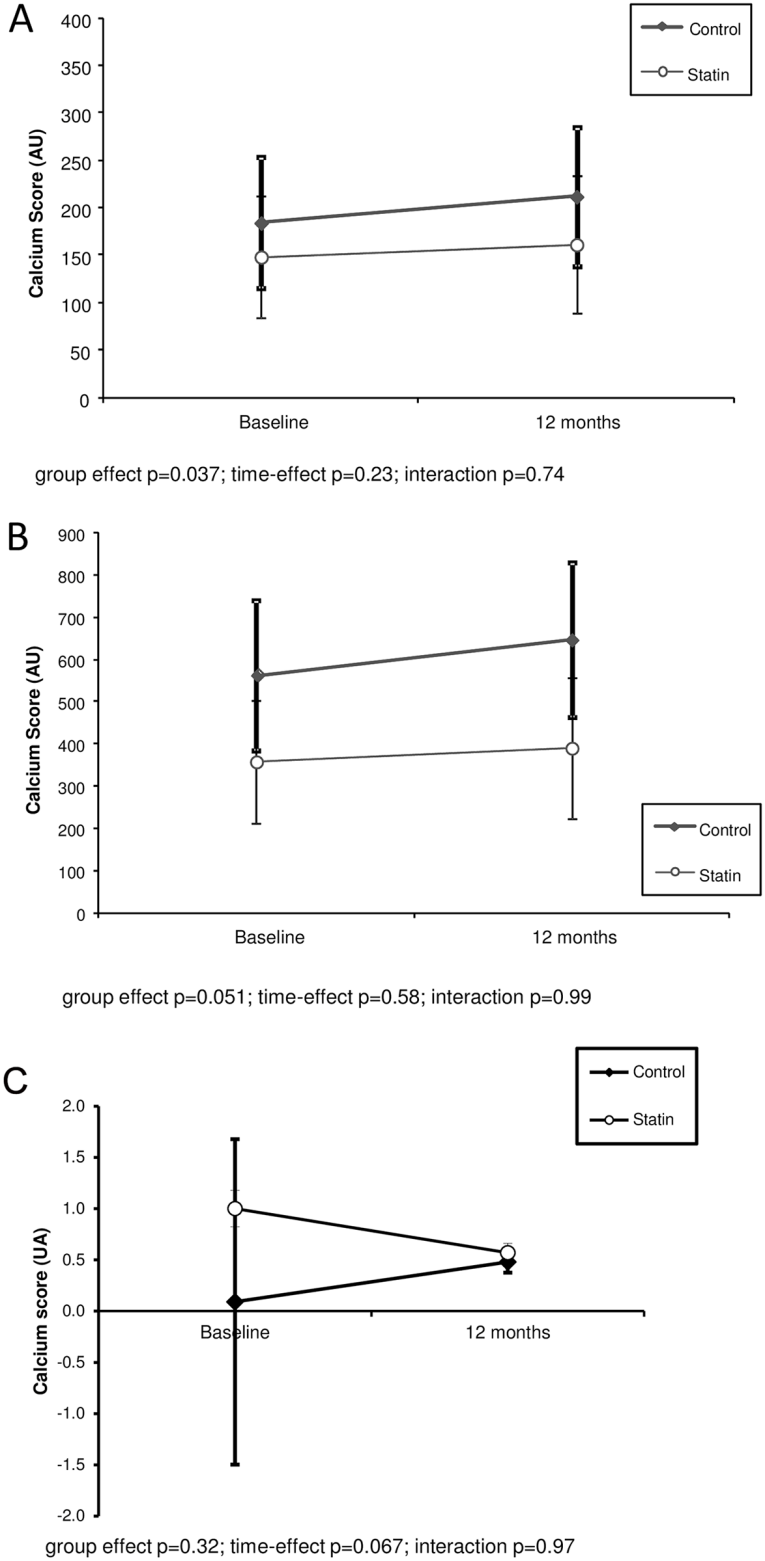
CAC progression in the groups (A—All patients; B—≥ 10 AU [calcified patients]; C—< 10 AU [non calcified patients]).

In the multivariate model (GEE), adjusting for gender, prior dialysis time, BMI, HDL-c, LDL-c, and triglycerides, there was no difference in CAC progression between the groups (group effect p = 0.037; time-effect p = 0.23; interaction p = 0.74). Similar results were obtained when only patients with CAC score ≥ 10AU (calcified) were analyzed (group effect p = 0.06; time-effect p = 0.70; interaction p = 0.92) (Fig 3). Of note, no difference was observed in the analyses excluding diabetic patients, considering the presence of hypertension and the effects of hypertensive drugs.

Seven patients had cardiovascular events during the follow-up, of whom two in statin group (one had coronary disease and other cerebrovascular disease) and five in control group (one had cerebrovascular disease, three deep venous thrombosis and one pulmonary thromboembolism). Three deaths occurred, only in the control group (one due to cardiovascular disease, one septic shock and one hypovolemic shock secondary to acute abdominal bleeding).

## Discussion

In the present study of incident kidney transplant recipients, the same extent of CAC progression across the statin and control groups was observed. The therapeutic intervention did not delay CAC progression at the 12-month follow up.

Similar to Seyahi *et al* [19], the present study demonstrated CAC progression in a relative young KTR with no history of coronary artery disease. Alike, a recent cohort had shown coronary calcification progression in KTRs, and the progression was independently associated with a higher baseline CAC score [20]. In contrast, some papers have shown that in incident KTRs, the progression of CAC can slow down or stabilize [21–23]. Possible explanations for these inconsistent findings are the differences in the study population characteristics and the definition of CAC progression.

It is well established that a reduction in LDL cholesterol leads to a decrease in cardiovascular event in the general population. In fact, major cardiovascular events were reduced by 22% with 41 mg/dL LDL reduction in the CTT study [24]. In the dialysis population, although 4D and AURORA studies had showed negative results [9,10], SHARP study [12] demonstrated a reduction in cardiovascular events by lowering LDL cholesterol. Additionally, in renal transplantation, ALERT study showed a 32% decrease in LDL cholesterol with significant impact on survival [11]. Moreover, a post hoc analysis of the ALERT showed a reduction in cardiovascular events in patients taking statin [25]. In the present study despite 27% reduction of LDL cholesterol, a similar reduction observed in these studies, an impact CAC progression was not observed. In kidney transplant scenario, it is important to consider the role of immunosuppressive regimen in lipid profile [26–28]. Some studies [26,27] have shown an increase in all lipid fractions related to immunosuppressive drugs in KTRs [28]. Of note these effects were dependent of type and dose of immunosuppressors [26,27]. In the present study there were no differences in lipid profile considering the immunosuppressive regimen.

The benefit of statin therapy on coronary calcification progression has been shown in the general population [29–30]. Some studies have demonstrated a relationship between the reduction in LDL cholesterol from a statin regimen and regression of coronary calcification [30–32]. In contrast, *Tenenbaum et al* in a *post hoc* analysis of the ACTION study showed that CAC progression was not associated with the long term treatment of LDL-c with statins [33]. A possible explanation for these conflicting findings could be the fact that CAC score does not show non-calcified plaques or the inflammatory status of atheroma. Recently, Tawakol *et al* [34] validated a positron emission tomography imaging and showed a reduction on vascular inflammation with statin therapy, despite a non-significant change in CAC score. A dose dependent effect of rosuvastatin in reducing atheroma volume, evaluated by intravascular ultrasound, was also observed in the ASTEROID trial [35]. On the other hand REVERSAL trial [36] showed a maintaining/progression in atheroma volume using atorvastatin. Of note, in the present study was observed a trend to a greater coronary calcification in the control group (p = 0.051) when we analyze only the patients with CAC > 10 (calcified). Although we cannot infer an effect of statins on CAC score over the time, since an interaction between the groups were not significantly (p = 0.999).

In agreement with non controlled studies we were also not able to demonstrate a decrease in inflammatory markers in patients using statins, however, since in the current study an increase in CRP levels was found in the control group during the follow-up, a potential beneficial effect in preventing the increase of inflammation in these patients could be speculated. The studies that could not demonstrate an effect of statins on inflammatory markers in KTRs had no control group [37,38]. Moreover, recently, Perez *et al* showed the anti-inflammatory action of statins in the KTR population. Although no change in CRP level was found, a decrease in bradykinin and complement C4 factor was observed [39]. Of note, increased inflammatory markers have been associated with cardiovascular events and mortality in renal transplant recipients [40].

Some studies have failed to demonstrate an improvement in graft function in renal transplant recipients receiving statin therapy [41,42]. In ALERT study, fluvastatin could not prevent a decline in renal function or graft loss [43]. However, other studies have demonstrated a significant benefit of statins on graft function [44–46]. In the present study, better renal graft function in the statin group compared to the control group was observed. Futhermore, this group had a smaller decreased of HDLc. Interestingly, Baragetti *et al*. showed that low HDLc levels were associated with reduced GFR [47]. A possible explanation for this benefit could be that the drug was introduced immediately after kidney transplantation, and the pleiotropic effects could be more pronounced during this phase. Of note, an elevated serum creatinine was a strong independent risk factor for CVD in KTRs [48].

To our knowledge, this study is the first to compare the effect of the early use of statins on CAC progression in incident KTRs. Although the statin dose used had been sufficient to lower LDL cholesterol significantly and in line with the results of CKD trials, the drug regimen of fixed-dose may have limited the potential benefits in this population. The anti-inflammatory actions of statins may depend on the dose administered, and most studies in a non-renal population were designed using higher doses of statins or over a longer period of time [49,50]. In dialysis patients, several studies have failed to find a benefit for statins on mortality using a relatively low dose [9, 10]. Recently a systematic review was not able to demonstrate a reduction of cardiovascular events with low doses of statin in KTR studies [51]. Therefore, an insufficient dose, relatively small and young sample, few diabetic subjects, short follow up duration and unequal baseline LDLc distribution between the groups could be potential reasons for the ineffectiveness of this treatment regimen on CAC progression and clinical outcomes. These factors should be considered the limitations of the present study.

We conclude that although statins reduce the levels of cholesterol and triglycerides, enhance graft function and diminish inflammation, the dose adopted in the current study was not able to delay CAC progression during 12 months of follow up. As CAC involves a complex pathway, we could hypothesize that CAC requires a multi-target drug approach, perhaps in higher doses and in a long-term manner to significantly halt CAC progression in early KTR. Moreover, a recent meta-analysis had suggested that the atherosclerotic coronary plaque regression were not associated with major cardiovascular events [52]. Therefore, further studies are necessary to confirm the impact of CAC regression and the role of statin in this population.

## Supporting Information

S1 CONSORT Checklist CONSORT Checklist. (DOC)

S1 Protocol Trial study protocol (English). (DOCX)

S2 Protocol Original trial protocol (Brasilian Portuguese). (DOC)
