# Supplementary material for: Effect of Statins on the Progression of Coronary Calcification in Kidney Transplant Recipients
Source: PLoS One. 2016 Apr 21;11(4):e0151797. doi: 10.1371/journal.pone.0151797 (PMC4839705; doi:10.1371/journal.pone.0151797)
Supplement: S1 Protocol — (DOCX) [file pone.0151797.s002.docx]

**TITLE:** The effects of statin on the progression of cardiovascular disease in kidney transplant patients

**1. INTRODUCTION**

The transplant is a widely used renal replacement therapy and has been associated with improved quality of life, lower cost, less morbidity and increased survival rate when compared to dialysis (1-4). Despite the kidney transplant is an effective therapy, the 5-year survival is 90.2% for patients with living donor transplantation and 81.1% for those with cadaver donor (5).

Cardiovascular complications are the leading cause of mortality (6), comprising 30-60% of deaths in this population (6). Acute coronary syndrome is the most common cardiovascular event in transplant, affecting about one in every 100 patient per year (7-10). Studies suggest that coronary heart disease is more aggressive in patients with deficit of renal function, as evidenced by the increased frequency, severity and more rapid progression of atherosclerotic lesions in these patients than in the general population (11).

Coronary calcification was considered a marker of atherosclerosis, were a common finding in patients with chronic kidney disease (CKD) (12,13). Some studies had showed that the extent of calcification is predictive of cardiovascular events and death (14,15). In previous studies of our group, using Multi-slice coronary tomography, it was observed that 79% in hemodialysis patients, 59% in peritoneal dialysis and 56% in conservative treatment (pre-dialysis) had some degree of calcification (16 -18). It is noteworthy that severe calcification, represented by calcium score above 400, were present in respectively 31%, 23% and 22% of patients on hemodialysis, peritoneal dialysis and conservative, indicating a high risk for cardiovascular events.

Few data are available on coronary calcification in patients after renal transplantation (19,20). If on one hand the successful transplant restores kidney function, a fact that may contribute to decrease the occurrence and progression of vascular calcification, on the other hand, these patients often hypertension, diabetes, dyslipidemia, obesity and smoking, known risk factors for atherosclerosis, to which is added the use of immunosuppressive drugs, the presence of inflammation, rejection, graft dysfunction (21,22), infection (23), proteinuria (24), hyperhomocysteinemia (25) and anemia (26), factors that They may also be associated with cardiovascular disease (CVD) in this population.

About these factors, inflammation stands out, which seems to be the main link between the traditional risk factors and non-traditional. The state of chronic inflammation was often observed in patients in different stages of CKD (27), as well as those on dialysis (28). In transplant patients the data are controversial. Some studies have shown a significant decrease in inflammatory markers in the first two months post-transplant in the absence of rejection (29). However, others studies have shown that despite the initial lowering levels of inflammatory markers, especially interleukin-6 and TNF, they are increased 12 months after transplantation (30).

The presence of inflammation has been linked to the loss of the renal graft and CVD (31,32). One possible explanation is that inflammation determines endothelial dysfunction, starting point of atherosclerosis (33). In fact, studies have shown the presence of endothelial dysfunction in kidney transplant patients, even those who had no risk factors for CVD (34). Furthermore, the inflammation decreases the action of lipases with consequent increase of LDL cholesterol (35). It is noteworthy that the oxidation of LDL has been implicated as the main mechanism of atherosclerosis (36).

Dislipidemia é um achado freqüente em pacientes transplantados. Em estudo multicêntrico observou-se que após um ano do transplante, 80 a 90% dos pacientes apresentavam concentrações de colesterol total > 200 mg/dL e 90 a 97% LDL > 100 mg/dL (37,38). As causas de dislipidemia após o transplante estão relacionadas à presença de síndrome nefrótica, disfunção do enxerto ou/e ao uso de hipotensores e imunossupressores principalmente corticoesteróides (39). O uso de tacrolimus tem sido associado à menores concentações dos níveis séricos de colesterol e triglicérides (40,41).

Another risxk factor that may contribute to cardiovascular disease in transplanted patients is the change in bone mineral metabolism. Similar to the dialysis patients, some studies suggest that changes in bone metabolism, especially the decrease in bone mass, are associated with mortality after transplantation (42).

Post-transplant bone disease is a complex situation and dependent on the pre-transplant renal osteodystrophy (43). Factors such as the partial re-stabilization of renal function and chronic administration of drugs that negatively influence on bone metabolism, especially corticosteroids, are the main causes of non-maintenance of post transplant osteodystrophy. Indeed, we observed a decrease of bone mass in post-transplant patients using glucocorticoids (44-46), data about another immunosuppressors are limited, but the use of tacrolimus are also appears to be associated with bone demineralization (47).

Another factor to consider is obesity, as transplant patients tend to gain weight (48,49). The increase of visceral fat has been associated with increased risk of CVD in the general population (50). This consequence may be related to the release of adipokines by adipose tissue (51). However the role of visceral fat in the CVD has not yet been proven in kidney transplant patients.

Some studies have shown that the use of statins in patients with CKD decreases cholesterol concentrations (52), and possibly attenuating endothelial dysfunction by its anti-inflammatory properties (53).

Despite not having been demonstrated increased survival of diabetic patients on dialysis with statin use (54), the ALERT study proved the effectiveness of these drugs in lowering cholesterol levels and the occurrence of cardiovascular events in transplant patients (55.56 ).

In summary, kidney transplant can control several factors related to the onset and progression of vascular calcification, moreover, it is also associated with several potentially modifiable atherogenic factors, such as dyslipidemia and inflammation. The use of statins can lead to a decrease in the progression of vascular calcification after renal transplantation with consequent improvement in survival in this population.

**2. OBJECTIVES**

. 2.1 PRIMARY:

* Evaluate the role of statins on the progression of cardiovascular disease in patients undergoing kidney transplantation

2.2 SECUNDARY:

- Identify the factors that contribute to the development or progression of cardiovascular disease in post kidney transplant
- To evaluate the relationship between cardiovascular disease and inflammation, bone metabolism, nutritional status and renal function
- To assess the effects of statins in post transplant renal function
- Analyze the effects of statins on cardiovascular events

**3. METODOLOGY**

Prospective, randomized, controlled, including patients with recent donor-vivo kidney transplant and regularly followed in the post-transplant clinic in Oswaldo Ramos, UNIFESP Foundation.

Number of selected patients: 150

Number of patients randomized: approximately 120

Patients will be randomized 1: 1 in statins and control groups.

Follow-up period: 12 months

**POPULATION:**

The selection will include patients in the immediate post-kidney transplant.

1. ***Inclusion criteria:***

- Use of calcineurin inhibitor as immunosuppressive treatment throughout the study selection periodhomem ou mulher com 18 a 65 anos
- Recent Postoperative renal (1-2 months)
- Estimated clearance of creatinine greater than 30 ml / min

2. ***Exclusion criteria:***

- patients with a formal indication for statin or fibrate
- patients with prescription of statin and fibrate within 3 months prior to transplantation
- cardiovascular event in the three months before renal transplantation
- patients with CHF functional class III or IV
- patients with severe hepatic impairment (Child C)

**LABORATORY EVALUATION:**

A whole blood sample will be collected fasting at baseline, 6 and 12 months for dosage: blood count, urea, creatinine, glucose, ALT, CPK, cholesterol, LDL, HDL and VLDL, triglycerides, blood level of tacrolimus or cyclosporine.

The same whole blood sample collected in fasting at baseline and after 12° months also be used for determination of: albumin, C-reactive protein, interleukin 6, venous blood gases, ionized calcium, phosphorus, alkaline phosphatase, PTH and 1, 25 (OH) 2 vitamin D.

It will be collected fasting at baseline, 6 and 12 months a urine sample to conduct examination of urine I.

Renal function is evaluated by formula CKD-EPI.

**CARDIOVASCULAR PARAMETERS:**

Cardiovascular parameters will be evaluated by echocardiography, multi-slice coronary **tomography** scan and evaluation of pulse wave velocity.

## Cardiovascular events will be recorded during the study through suggestive history, myocardial necrosis markers, ECG, echocardiography, myocardial scintigraphy and / or coronary coronary angiography.

## *Echocardiogram*

All patients underwent echocardiography at baseline and after 12 months of study entry. These exams will consist of evaluations with M-mode, two-dimensional and Doppler using Philips HDI 5000 (Philips Electronics, Netherlands). All analyzes will be carried out in accordance with the recommendations of the American Society of Echocardiography.

***Coronary tomography***

Coronary tomography will be held in Brazil Diagnostic Center with LightSpeed Pro16 equipment (GE Healthcare, Milwaukee, USA) at baseline and after 12 months of study entry. The calculation of calcium score is based on formulas that use volume measurements, density and area of the lesions, and expressed in units Agatston modified.

***Pulse wave velocity***

At baseline, 6 and 12 months will be obtained pulse wave velocities of the carotid and femoral arteries by a Complior®SP equipment (Artech Medical, Pantin, France) and analyzed by appropriate software.

***clinical variables***

Acute rejection was can defined as an acute deterioration of renal function, which is associated with specific pathological changes of the graft.

Chronic allograft nephropathy is a diagnosis suggested by the clinical picture which usually corresponds to a slow and gradual increase in serum creatinine, appearance / increase in proteinuria and worsening of blood pressure control. Usually a biopsy of the graft is performed and provides the degree and staging of renal impairment.

Cardiovascular disease wera defined as a spectrum of diseases ranging coronary heart disease, cardiomyopathy, heart valve disease, arrhythmia, cerebrovascular disease or peripheral vascular disease. Some or all of these entities can coexist or advancing in sequence over time.

Waist circumference is the most reliable anthropometric index of intra-abdominal fat. It is the measurement of the circumference of the abdomen in the middle of the distance between the iliac crest and the lower costal margin. It is recommended that this value be below 94 cm in men and 80 cm in women.

Body mass index (BMI) is used to estimate normal weight, overweight, obesity or malnutrition in the individual. It is obtained by dividing weight by height squared. Values between 25 kg / m² and 29.9 kg / m² define overweight and values above 30 kg / m² define obesity.

**4. TRATMENT**

Rosuvastatin is administered at a dose of 10mg once daily in the corresponding group.

All patients will be treated with immunosuppressive agents according to pre-established by the Foundation Osvaldo Ramos and adjusted the dose according to blood level of tacrolimus protocols.

Hypertensive patients was treated with calcium channel blockers, beta-blockers, converting enzyme inhibitors, diuretics and other hypotensive classes, according to the blood pressure control.

         Patients with hemoglobin less than 11 g / dL, receive recombinant human erythropoietin, after being away another cause of anemia and verified iron stores. The iron deficient patients (ferritin <100 ng / mL and / or transferrin saturation <20%) will receive intravenous iron supplementation (Noripurum 200mg / month).

Patients with hyperphosphatemia (P> 4,6mg / dl) will make use of binders in accordance with the recommendations of DOKQI / ASN.

**5. CRONOGRAMA DO ESTUDO**

|  | Selection | Baseline  (1-2 months) | 6 ^o^. months | 12 ^o^. months |
| --- | --- | --- | --- | --- |
| **informed consent** | X |  |  |  |
| **Criteria inclusion / exclusion** | X |  |  |  |
| **history** |  | X | X | X |
| **physical examination** |  | X | X | X |
| **cardiovascular events** |  | X | X | X |
| **Waist circumference and BMI** |  | X | X | X |
| **Laboratory examination** |  |  |  |  |
| Venous blood gases, Cai, P, AF, PTH, 1,25OHVitD, inflammatory markers, urine I |  | X |  | X |
| Blood count, cholesterol, kidney function, tacrolimus or cyclosporine, blood glucose, ALT, CPK |  | X | X | X |
| **Echocardiogram** |  | X |  | X |
| **Coronary tomography** |  | X |  | X |
| **Pulse wave velocity** |  | X | X | X |
| **Adverse events** |  |  | X | X |

**7. REFERENCES**

1. Laupacis A, Keown P, Pus N, et al: A study of the quality of life and cost-utility of renal transplantation. Kidney Int 1996; 50: 235
2. Russell JD, Beegcroft ML, Ludwin D, et al: The quality of life in renal transplantation – a prospective study. Transplantation 1992; 54: 656
3. Wolfe RA, Ashby VB, Milford EL, et al: Comparison of mortality in all patients on dialysis, patients on dialysis awaiting transplantation, and recipients of a first cadaveric transplant. N Engl J Med 1999; 341: 1725
4. Arend SM, Mallat MJ, Westendorp RJ, et al: Patient survival after renal transplantation more than 25 years follow-up. Nephrol Dial Transplant 1997; 12: 1672
5. HHS/HRSA/HSB/DOT. 2005 OPTN/SRTR Annual Report 1995-2004. www.ustransplant.org
6. Collins AJ, Kasiske B, Herzog C, Chavers B, Foley R, Gilbertson D, Grimm R, Liu J, Louis T, Manning W, Matas A, McBean M, Murray A, St. Peter W, Xue J, Fan Q, Guo H, Li S, Li S, Roberts T, Snyder J, Solid C, Wang C, Weinhandl E, Arko C, Chen SC, Dalleska F, Daniels F, Dunning S, Ebben J, Frazier E, Johnson R, Sheets D, Forrest B, Berrini D, Constantini E, Everson S, Frederick P, Eggers P, Agodoa L: Excerpts from the United States Renal Data System 2004 annual data report: Atlas of end stage renal disease in the United States Am J Kidney Dis 2005; 45(Suppl 1):A1
7. Rabbat CG, Thorpe KE, Russell JD, et al: Comparison of mortality risk for dialysis patients and cadaveric first renal transplant recipients in Ontário, Canadá. J Am Soc Nephrol 2000; 11: 917
8. West M, Sutherland DE, Matas AJ: Kidney transplant recipients who die with functioning grafts: serum creatinine level and cause of death. Transplantation 1996; 62: 1029
9. Lindholm A, Albrechtsen D, Frodin L, et al: Ischemic heart disease – major cause of death and graft loss after renal transplantation in Scandinavia. Transplantation 1995; 60: 451
10. Yeo FE, Villines TC, Bucci JR, et al: Cardiovascular risk in stage 4 and 5 hephrophaty. Adv Chronic Kidney Dis 2004; 11: 116
11. Braun J, Oldendorf M: EBCT in the evaluation of cardiac calcifications in chronic dialysis patients. Am J Kidney Dis 1996; 27: 394
12. Goodman WG, Goldin J, Kuizon BD, Yoon C, Gales B, Sider D, Wang Y, Chung J, Emerick A, Greaser L, Elashoff RM, Salusky IB: Coronary-artery calcification in young adults with end-stage renal disease who are undergoing dialysis. N Engl J Med 2000; 342: 1478
13. Russo D, [Palmiero G](http://www.ncbi.nlm.nih.gov/entrez/query.fcgi?db=pubmed&cmd=Search&itool=pubmed_AbstractPlus&term=%22Palmiero+G%22%5BAuthor%5D), [De Blasio AP](http://www.ncbi.nlm.nih.gov/entrez/query.fcgi?db=pubmed&cmd=Search&itool=pubmed_AbstractPlus&term=%22De+Blasio+AP%22%5BAuthor%5D), [Balletta MM](http://www.ncbi.nlm.nih.gov/entrez/query.fcgi?db=pubmed&cmd=Search&itool=pubmed_AbstractPlus&term=%22Balletta+MM%22%5BAuthor%5D), [Andreucci VE](http://www.ncbi.nlm.nih.gov/entrez/query.fcgi?db=pubmed&cmd=Search&itool=pubmed_AbstractPlus&term=%22Andreucci+VE%22%5BAuthor%5D): Coronary artery calcification in patients with CRF not undergoing dialysis. Am J Kidney Dis 2004; 44(6): 1024
14. Raggi P, Boulay A, Chasan-Taber S, Amin N, Dillon M, Burke S K, Chertow GM: Cardiac calcification in adult hemodialysis patients: a link between end-stage renal disease and cardiovascular disease? J Am Coll Cardiol 2002; 39 (4): 695
15. Matsuoka M, [Iseki K](http://www.ncbi.nlm.nih.gov/entrez/query.fcgi?db=pubmed&cmd=Search&itool=pubmed_AbstractPlus&term=%22Iseki+K%22%5BAuthor%5D), [Tamashiro M](http://www.ncbi.nlm.nih.gov/entrez/query.fcgi?db=pubmed&cmd=Search&itool=pubmed_AbstractPlus&term=%22Tamashiro+M%22%5BAuthor%5D), [Fujimoto N](http://www.ncbi.nlm.nih.gov/entrez/query.fcgi?db=pubmed&cmd=Search&itool=pubmed_AbstractPlus&term=%22Fujimoto+N%22%5BAuthor%5D), [Higa N](http://www.ncbi.nlm.nih.gov/entrez/query.fcgi?db=pubmed&cmd=Search&itool=pubmed_AbstractPlus&term=%22Higa+N%22%5BAuthor%5D), [Touma T](http://www.ncbi.nlm.nih.gov/entrez/query.fcgi?db=pubmed&cmd=Search&itool=pubmed_AbstractPlus&term=%22Touma+T%22%5BAuthor%5D), [Takishita S](http://www.ncbi.nlm.nih.gov/entrez/query.fcgi?db=pubmed&cmd=Search&itool=pubmed_AbstractPlus&term=%22Takishita+S%22%5BAuthor%5D): Impact of high coronary artery calcification score (CACS) on survival in patients on chronic hemodialysis. Clin Exp Nephrol 2004; 8(1): 54
16. Ammirati AL, Dalboni MA, Cendoroglo Neto M, Draibe SA, Canziani MEF: Coronary artery calcification, systemic inflammation markers and mineral metabolism in a peritoneal dialysis population”. Nephron Clin Pract 2006; 104 (1): 33
17. Barreto DV, Barreto FC, Carvalho AB, Cuppari L, Cendoroglo Neto M, Draibe SA, Moyses RMA, Neves KR, Jorgetti V, Blair A, Guiberteau R, Canziani MEF: Coronary calcification in hemodialysis patients: the contribution of traditional and uremia-related risk factors. Kidney Int 2005; 67: 1576
18. Tomiyama C, Higa A, Dalboni MA, Cendoroglo Neto M, Draibe SA, Cuppari L, Carvalho AB, Neto EM, Canziani ME: The impact of traditional and non-traditional risk factors on coronary calcification in pre-dialysis patients. Nephrol Dial Transplant 2006; 21: 2467
19. Moe SM, O’Niel KD, Resterova M, Fineberg N, Persohn S, Meyer CA: Natural history of vascular calcification in dialysis and transplant patients. Nephrol Dial Transplant 2004; 19: 2387
20. Oschatz E, Benesch T, Kodras K, Hoffmann U, Haas M: Changes of coronary calcification after kidney transplantation. Am J Kidney Dis 2006; 48: 307
21. Abbott KC, Bucci JR, Cruess D, et al: Graft loss and acute coronary syndromes after renal transplantation in the United States. J Am Soc Nephrol 2002; 13: 2560
22. Duclox D, Kazory A, CHalopin JM. Predicting coronary heart disease in renal transplant recipients: A prospective study. Kidney Int 2004; 66: 441
23. Humar A, Gillingham K, Payne WD, Sutherland DER, Matas AJ: Increased incidence of cardiac complications in kidney transplant recipients with cytomegalovirus disease. Transplantation 2000; 70(2): 310
24. Sarnak MJ, Levey AS. Cardiovascular disease and chronic renal disease: a new paradigm. Am J Kidney Dis 2000; 35 (Suppl. 1): S117
25. Arnadottir M, Hultberg B, Wahlberg J, et al: Serum total homocysteine concentration before and after renal transplantation. Kidney Int 1998; 54: 1380
26. Mix TCH, Pereira JGP, Kausz AT, et al: Anemia: A continuing problem following kidney transplantation. Am J Transplant 2003; 3: 1426
27. Romão JE Júnior, Haiashi AR, Elias RM, Luders C, Ferraboli R, Castro MC, Abensur H: Positive acute-phase inflammatory markers in different stages of chronic kidney disease. Am J Nephrol 2006; 26(1): 59
28. Stenvinkel P, Ketteler M, Johnson RJ, et al. IL-10, IL-6, and TNF-alpha: central factors in the altered cytokine network of uremia – the good, the bad, and the ugly. Kidney Int2005; 67:1216
29. Simmons EM, Langone A, Sezer MT, Vella JP, Recupero P, Morow JD, Ikizler TA, Himmelfarb J: Effect of renal transplantation on biomarkers of inflammation and oxidative stress in end-stage renal disease patients. Transplantation 2005; 79(8): 914
30. Cueto-Manzano AM, Morales-Buenrostro LE, Gonzáles-Espinosa L, Gonzáles-Tabaleros N, Martín-del-Campo F, Correa-Rotter R, Valera I, Alberú J: Markers of inflammation before and after renal transplantation. Transplantation 2005; 80 (1): 47
31. Ridker PM, Cushman M, Stampfer MJ, Tracy RP, Hennekens CH: Inflammation, aspirin, and the risk of cardiovascular disease in apparently healthy men. N Engl J Med 1997; 336: 973
32. Bakri RS, Afzali B, Covic A *et al*: Cardiovascular disease in renal allograft recipients is associated with elevated sialic acid or markers of inflammation. Clin Transplant 2004; 18: 201
33. Ross R: Atherosclerosis — An inflammatory disease. N Engl J Med 1999; 340(2): 115
34. Kocak H, Ceken K, Dinckan A, Mahsereci E, Yavuz A, Yucetin L, Akbas S, Gurkan A, Erdogan O, Ersoy F: Assessment and comparison of endothelial function between dialysis and kidney transplant patients. Transplantation Proc. 2006; 38(2): 416
35. Davignon J,Ganz P: Role of Endothelial Dysfunction in Atherosclerosis. Circulation 2004; 109: III-27
36. Steinberg D, Witztum JL. Is the oxidative modification hypothesis relevant to human atherosclerosis? Circulation 2002; 105: 2107
37. Gonyea JE, Anderson CF: Weight change and serum lipoproteins in recipients of renal allografts. Mayo Clin Proc 1992; 67: 653
38. Moore R, Thomas D, Morgan E, et al: Abnormal lipid and lipoproteins profiles following renal transplantation: Tranplant proc 1993; 25: 1060
39. Vanrenterghem Y, et al: Double-blind comparison of two corticosteroid regimens plus mycophenolate mofetil and cyclosporine for prevention of acute renal allograft rejection. Transplantation 2000; 70: 1352
40. Artz MA, et al: Improved cardiovascular risk profile and renal function in renal transplant patients after conversion from cyclosporine to tacrolimus. J Am Soc Nephrol 2003; 14: 1880
41. Kramer BK, Montagnino G, Del Castillo D, et al: Efficacy and safety of tacrolimus compared with cyclosporin A microemulsion in renal transplantation: 2 year follow-up results. Nephrol Dial Transplant 2005; 20: 968
42. Heaf J, Tvedegaard E, Kanstrup IL, Fogh-Andersen N: Hyperparathyroidism and long-term bone loss after renal transplantation. Clin Transplant 2003; 17: 268
43. Bellorin-Font E, Rojas E, Carlini RG, Suniaga O, Weisinger JR: Bone remodeling after renal transplantation. Kidney Int 2003; 85 (suppl): S125
44. Julian BA, Laskow DA, Dubovsky J, Dubovsky EV, Curtis JJ, Quarles LD: Rapid loss of vertebral mineral density after renal transplantation. N Engl J Med 1991; 325: 544
45. Monier-Faugere MC, Mawad H, Qi Q, Friedler RM, Malluche HH: High prevalence of low bone turnover and occurrence of osteomalacia after kidney transplantation. J Am Soc Nephrol 2000; 11: 1093
46. Almond MK, Kwan JT, Evans K, Cunningham J: Loss of regional bone mineral density in the first 12 months following renal transplantation. Nephron 1994; 66: 52
47. Epstein S: Post-transplantation bone disease: The role of immunosuppressive agents and the skeleton. J Bone Miner Res 1996; 11:1
48. **Baum C:** Weight gain and cardiovascular risk after organ transplantation. Journal of Parenteral and Enteral Nutrition. 2001; 25(3): 114
49. El-Agroudy AE, Wafa EW, Gheith OE, Shehab El-Dein AB, Ghoneim MA: Weight gain after renal transplantation is a risk factor for patient and graft outcome. Transplantation. 2004; 77(9):1381
50. Despres JP: Intra-abdominal obesity: an untreated risk factor for Typ2 diabetes and cardiovascular disease. J Endocrinol Invest 2006; 29(3 Suppl): 77
51. [Lau DC, Dhillon B, Yan H, Szmitko PE, Verma S](http://www.ncbi.nlm.nih.gov/entrez/query.fcgi?db=pubmed&cmd=Retrieve&dopt=AbstractPlus&list_uids=15653761&query_hl=9&itool=pubmed_docsum): Adipokines: molecular links between obesity and atheroslcerosis. Am J Physiol Heart Circ Physiol. 2005; 288(5):H2031
52. Seliger SL, Weiss NS, Gillen DL, Kestenbaum B, Ball A, Sherrard DJ, Stehman-Breen CO: HMG-CoA reductase inhibitors are associated with reduced mortality in ESRD patients. Kidney Int. 2002; 61(1):297
53. Massy ZA, Guijarro C: Statins: effects beyond cholesterol lowering Nephrol. Dial. Transplant. 2001; 16(9): 1738
54. [Wanner C, Krane V, Marz W, Olschewski M, Mann JF, Ruf G, Ritz E: German Diabetes and Dialysis Study Investigators.](http://www.ncbi.nlm.nih.gov/entrez/query.fcgi?db=pubmed&cmd=Retrieve&dopt=AbstractPlus&list_uids=16034009&query_hl=5&itool=pubmed_docsum) Atorvastatin in patients with type 2 diabetes mellitus undergoing hemodialysis. N Engl J Med 2005; 353(3):238
55. Åsberg A, Hartmann A, Fjeldså E, Holdaas H: Atorvastatin improves endothelial function in renal-transplant recipients. Nephrol Dial Transplant 2001; 16: 1920
56. Holdaas H, Fellström B, Cole E, Nyberg G, Olsson AG, Pedersen TR, Madsen S, Grönhagen-Riska C, Neumayer H-H, Mães B, Ambühl P, Hartmann A, Staffler B, Jardine AG: Long-term Cardiac Outcomes in Renal Transplant Recipients Receiving Fluvastatin: The ALERT Extension Study. Am J Transplant 2005; 5(12): 2929
