# Supplementary material for: Effect of Statins on the Progression of Coronary Calcification in Kidney Transplant Recipients
Source: PLoS One. 2016 Apr 21;11(4):e0151797. doi: 10.1371/journal.pone.0151797 (PMC4839705; doi:10.1371/journal.pone.0151797)
Supplement: S2 Protocol — (DOC) [file pone.0151797.s003.doc]

**TÍTULO :** Os efeitos da Estatina na progressão da doença cardiovascular em pacientes transplantados renais

**1. INTRODUÇÃO**

O transplante é uma terapia de substituição renal amplamente utilizado e tem sido associado a melhor qualidade de vida, menor custo, menor morbidade e maior sobrevida quando comparado a diálise (1-4). Apesar do transplante renal ser uma terapia eficiente, a sobrevida em 5 anos é de 90,2% para pacientes com transplante de doador vivo e de 81,1% para aqueles com doador cadáver (5).

As complicações cardiovasculares constituem a principal causa de mortalidade (6), perfazendo 30 a 60% dos óbitos nessa população (6). A síndrome coronariana aguda é o evento cardiovascular mais comum em transplantados, atingindo aproximadamente um em cada 100 paciente.ano (7-10). Estudos sugerem que a doença coronária é mais agressiva em pacientes com déficit de função renal, fato evidenciado pela maior freqüência, severidade e progressão mais rápida das lesões ateroscleróticas nesses pacientes em comparação a população geral (11).

Calcificação coronária considerada um marcador de aterosclerose, é um achado comum em pacientes com doença renal crônica (DRC) (12,13). Alguns estudos demonstraram que a extensão da calcificação é preditiva de ocorrência de eventos cardiovasculares e óbito (14,15). Em estudos anteriores do nosso grupo, utilizando tomografia coronariana Multi-slice, observou-se que 79% dos pacientes em hemodiálise, 59% dos em diálise peritonial e 56% dos em tratamento conservador (pré-diálise) apresentaram algum grau de calcificação (16-18). Vale ressaltar, que calcificações severas, representadas por escore de cálcio acima de 400, estiveram presentes em respectivamente 31%, 23% e 22% dos pacientes em hemodiálise, diálise peritonial e conservador, indicando alto risco para ocorrência de eventos cardiovasculares.

Poucos dados são disponíveis sobre calcificação coronariana em pacientes após o transplante renal (19,20). Se por um lado o transplante bem sucedido restabelece função renal, fato esse que pode contribuir para diminuição da ocorrência e progressão da calcificação vascular, por outro lado, esses pacientes apresentam freqüentemente hipertensão arterial, diabetes, dislipidemia, obesidade e tabagismo, reconhecidos fatores de risco para aterosclerose, aos quais se somam o uso de imunossupressores, a presença de inflamação, rejeição, disfunção do enxerto (21,22), infecção (23), proteinúria (24), hiperhomocisteinemia (25) e anemia (26), fatores que também podem estar associados a doença cardiovascular (DCV) nessa população.

Dentre esses fatores destaca-se a inflamação, que parece ser o principal elo de ligação entre os fatores de risco tradicionais e os não tradicionais. O estado de inflamação crônica é freqüentemente observado em pacientes nos diferentes estágios da DRC (27), assim como naqueles em diálise (28). Em pacientes transplantados os dados são controversos. Alguns estudos mostraram uma diminuição significativa dos marcadores inflamatórios nos primeiros dois meses pós-transplante na ausência de rejeição (29). Entretanto, outros mostraram que apesar da diminuição inicial, os marcadores inflamatórios, principalmente a interleucina 6 e TNF, encontram-se aumentados após 12 meses do transplante (30).

A presença de inflamação nessa população tem sido associada à perda do enxerto e a ocorrência de DCV (31,32). Uma possível explicação seria a de que a inflamação determina disfunção endotelial, marco inicial da aterosclerose (33). De fato, estudos demonstram a presença de disfunção endotelial em transplantados renais, mesmo naqueles que não apresentavam fatores de risco para DCV (34). Além disso, diminui a ação das lípases com conseqüente aumento do LDL colesterol (35). Vale ressaltar, que a oxidação do LDL tem sido implicada como o principal mecanismo do processo aterosclerótico (36).

Dislipidemia é um achado freqüente em pacientes transplantados. Em estudo multicêntrico observou-se que após um ano do transplante, 80 a 90% dos pacientes apresentavam concentrações de colesterol total > 200 mg/dL e 90 a 97% LDL > 100 mg/dL (37,38). As causas de dislipidemia após o transplante estão relacionadas à presença de síndrome nefrótica, disfunção do enxerto ou/e ao uso de hipotensores e imunossupressores principalmente corticoesteróides (39). O uso de tacrolimus tem sido associado à menores concentações dos níveis séricos de colesterol e triglicérides (40,41).

Outro fator que pode contribuir para doença cardiovascular nos pacientes transplantados é a alteração do metabolismo mineral ósseo. A exemplo do que acontece com pacientes em diálise, alguns estudos sugerem que as alterações do metabolismo mineral ósseo, principalmente a diminuição da massa óssea, estão associadas à mortalidade após o transplante (42).

A doença óssea pós-transplante é uma situação complexa e dependente da osteodistrofia renal pré-transplante (43). Fatores como a re-estabilização parcial da função renal e administração crônica de drogas que influenciam negativamente com o metabolismo ósseo, principalmente corticóides, são as principais causas da não manutenção da osteodistrofia renal no pós transplante. De fato, observou-se uma diminuição de massa óssea no pós-transplante em pacientes utilizando glicocorticóides (44-46), dados sobre outros imunossupressores são limitados, porém o uso do tacrolimus também parece estar associado a desmineralização óssea (47).

Um outro fator a ser considerado é a obesidade, visto que pacientes transplantados tendem a ganhar peso (48,49). O aumento de gordura visceral abdominal tem sido associado ao aumento do risco de DCV na população geral (50). Esse fato pode estar relacionado à liberação pelo tecido adiposo de adipocinas (51). Entretanto o papel da gordura visceral na DCV ainda não foi comprovado em pacientes transplantados renais.

Alguns estudos têm demonstrado que a utilização de estatinas em pacientes com DRC diminui as concentrações de colesterol (52), alem de atenuar a disfunção endotelial possivelmente por suas propriedades anti-inflamatórias (53). Apesar de não ter sido demonstrado aumento da sobrevida de pacientes diabéticos submetidos à diálise com uso de estatina (54), o estudo ALERT comprovou a eficácia dessas drogas em diminuir os níveis de colesterol e a ocorrência de eventos cardiovasculares em pacientes transplantados (55,56).

Em resumo, se por um lado o transplante pode controlar vários fatores relacionados ao aparecimento e a progressão da calcificação vascular, por outro lado, também se associa a vários fatores aterogênicos potencialmente modificáveis, tais como a dislipidemia e a inflamação. O uso de estatina pode levar a uma diminuição da progressão da calcificação vascular após o transplante renal com conseqüente melhora da sobrevida nessa população.

**2. OBJETIVOS**

2.1 PRIMÁRIO:

* Avaliar o papel da estatina na progressão da doença cardiovascular em pacientes submetidos a transplante renal

2.2 SECUNDÁRIOS:

- Identificar os fatores que contribuem para o desenvolvimento ou progressão da doença cardiovascular no pós transplante renal
- Avaliar a relação entre doença cardiovascular e inflamação, metabolismo ósseo, estado nutricional e função renal
- Avaliar os efeitos da estatina na função renal pós transplante
- Analisar os efeitos da estatina na ocorrência de eventos cardiovasculares

**3. METODOLOGIA**

Estudo prospectivo, randomizado, controlado, incluindo pacientes recém transplantados renais doadores-vivo e regularmente seguidos no ambulatório de pós-transplante na Fundação Oswaldo Ramos, UNIFESP.

Número de pacientes selecionados: 150

Número de pacientes randomizados: aproximadamente 120

Os pacientes serão randomizados 1:1 nos grupos estatina e controle .

Tempo de seguimento: 12 meses

**POPULAÇÃO:**

A seleção incluirá pacientes no pós-transplante renal imediato.

1. ***Critérios de inclusão***:

- Uso de inibidor de calcineurina como tratamento imunossupressor durante todo o período de seleção do estudo
- homem ou mulher com 18 a 65 anos
- Pós-operatório recente de transplante renal (1-2 meses)
- Clearence de creatinina maior que 30 ml/minuto

2. ***Critérios de exclusão:***

- pacientes com indicação formal de estatina ou fibratos
- pacientes com uso de estatina ou fibrato nos 3 meses anteriores ao transplante
- evento cardiovascular nos três meses que antecedem o transplante renal
- pacientes com ICC classe funcional III ou IV
- pacientes com insuficiência hepática grave (Child C)

**AVALIAÇÃO LABORATORIAL:**

Uma amostra de sangue total será coletado em jejum no início do estudo, 6° e 12° meses para dosagem de: hemograma, uréia, creatinina, glicemia, TGP, CPK , colesterol, LDL, HDL e VLDL, triglicerídeos, nível sanguíneo de tacrolimus ou ciclosporina.

A mesma amostra de sangue total coletado em jejum no início do estudo e após 12° meses também será utilizada para dosagem de: albumina, proteína-C reativa, interleucina 6, gasometria venosa, cálcio iônico, fósforo, fosfatase alcalina, PTH e 1,25 (OH)_2_ vitamina D.

Será coletado em jejum no início do estudo, 6° e 12° meses uma amostra de urina para realização de exame de Urina I.

A função renal será avaliada através da formula de CKD-EPI.

**PARÂMETROS CARDIOVASCULARES:**

Os parâmetros cardiovasculares serão avaliados através de ecocardiograma, tomografia coronariana multi-slice e avaliação da velocidade de onda de pulso.

Eventos cardiovasculares serão registrados durante o estudo através de história sugestiva, marcadores de necrose miocárdica, ECG, ecocardiograma, cintilografia miocárdica e/ou cineangiografia coronária.

## *Ecocardiograma*

Todos os pacientes realizarão ecocardiograma no início e após 12 meses do início do estudo. Esses exames constarão de avaliações com M-mode, bi-dimensional e Doppler utilizando equipamento Philips HDI 5000 (Royal Philips Electronics, Netherlands). Todas as análises serão realizadas de acordo com as recomendações da Sociedade Americana de Ecocardiografia.

***Tomografia Coronariana***

A tomografia coronariana será realizada no Centro de diagnóstico do Brasil com equipamento LightSpeed Pro16 (GE Healthcare, Milwaukee, USA) no início e após 12 meses do início do estudo. O cálculo do escore de cálcio será baseado em fórmulas que utilizam as medidas do volume, área e densidade das lesões, e expresso em unidades Agatston modificadas.

***Velocidade de onda de pulso***

No início do estudo, 6° e 12° meses serão obtidas as velocidades de ondas de pulso das artérias carótidas e femurais por um equipamento Complior®SP (Artech Medical, Pantin, France) e analisadas por software apropriado.

***Variáveis clínicas***

Rejeição aguda pode ser definida como uma deterioração aguda da função renal, que é associada a alterações patológicas específicas do enxerto.

Nefropatia crônica do enxerto é um diagnóstico sugerido pelo quadro clínico que usualmente corresponde a um lento e gradativo aumento da creatinina sérica, aparecimento/aumento da proteinúria e piora do controle pressórico. Geralmente uma biopsia do enxerto é realizada e nos fornece o grau e estadiamento do comprometimento da função renal.

Doença cardiovascular é definida como um espectro de doenças que abrangem a doença coronariana, miocardiopatia, doença valvar cardíaca, arrtimia, doença cerebrovascular ou doença vascular periférica. Algumas ou todas essas entidades podem coexistir ou progredirem em seqüência ao longo do tempo.

A circunferência abdominal é o índice antropométrico mais fidedigno de gordura intra-abdominal. É a medida da circunferência do abdômen no meio da distância entre a crista ilíaca e o rebordo costal inferior. Recomenda-se que esse valor seja abaixo de 94 cm em homens e de 80 cm em mulheres

Índice de massa corpórea (IMC) é usado para se estimar peso normal, sobrepeso, obesidade ou desnutrição no indivíduo. É obtido através da divisão do peso pela altura ao quadrado. Valores entre 25 Kg/m² e 29,9 Kg/m² definem sobrepeso e valores acima de 30 Kg/m² definem obesidade.

**4. TRATAMENTO**

A Rosuvastatina será administrada na dose de 10mg uma vez ao dia no grupo correspondente.

Todos os pacientes serão tratados com Imunossupressores de acordo com os protocolos pré-estabelecidos pela Fundação Osvaldo Ramos e reajustado a dose de acordo com nível sanguíneo do tacrolimus.

Os pacientes hipertensos serão tratados com bloqueadores de canais de cálcio, beta-bloqueadores, inibidores de enzimas de conversão, diuréticos ou outras classes de hipotensores, de acordo com o controle pressórico.

Os pacientes com hemoglobina inferiores a 11 g/dL, receberão eritropoetina recombinante humana, após ser afastada outra causa de anemia e verificado os estoques de ferro. Os pacientes com deficiência de ferro (ferritina <100ng/mL e/ou saturação de transferrina <20%) receberão suplementação de ferro endovenosa (Noripurum® 200mg/mês).

Pacientes com hiperfosfatemia (P>4,6mg/dl) farão uso de quelantes de acordo com as recomendações do DOKQI/ASN.

**5. CRONOGRAMA DO ESTUDO**

|  | Seleção | Início  (1-2 meses) | 6 ^o^.  mês | 12 ^o^. mês |
| --- | --- | --- | --- | --- |
| **Consentimento informado** | X |  |  |  |
| **Critérios inclusão/exclusão** | X |  |  |  |
| **História** |  | X | X | X |
| **Exame físico** |  | X | X | X |
| **Eventos cardiovasculares** |  | X | X | X |
| **Cintura abdominal e IMC** |  | X | X | X |
| **Ex Laboratorial** |  |  |  |  |
| Gaso venosa, Cai, P, FA, PTH, 1,25OHVitD, Marcadores inflamatórios, Urina I |  | X |  | X |
| Hemograma, CTF, função renal, FK ou ciclosporina, glicemia, TGP, CPK |  | X | X | X |
| **Ecocardiograma** |  | X |  | X |
| **Tomografia coronariana** |  | X |  | X |
| **Velocidade de onda pulso** |  | X | X | X |
| **Eventos adversos** |  |  | X | X |

**7. REFERÊNCIAS**

1. Laupacis A, Keown P, Pus N, et al: A study of the quality of life and cost-utility of renal transplantation. Kidney Int 1996; 50: 235
2. Russell JD, Beegcroft ML, Ludwin D, et al: The quality of life in renal transplantation – a prospective study. Transplantation 1992; 54: 656
3. Wolfe RA, Ashby VB, Milford EL, et al: Comparison of mortality in all patients on dialysis, patients on dialysis awaiting transplantation, and recipients of a first cadaveric transplant. N Engl J Med 1999; 341: 1725
4. Arend SM, Mallat MJ, Westendorp RJ, et al: Patient survival after renal transplantation more than 25 years follow-up. Nephrol Dial Transplant 1997; 12: 1672
5. HHS/HRSA/HSB/DOT. 2005 OPTN/SRTR Annual Report 1995-2004. www.ustransplant.org
6. Collins AJ, Kasiske B, Herzog C, Chavers B, Foley R, Gilbertson D, Grimm R, Liu J, Louis T, Manning W, Matas A, McBean M, Murray A, St. Peter W, Xue J, Fan Q, Guo H, Li S, Li S, Roberts T, Snyder J, Solid C, Wang C, Weinhandl E, Arko C, Chen SC, Dalleska F, Daniels F, Dunning S, Ebben J, Frazier E, Johnson R, Sheets D, Forrest B, Berrini D, Constantini E, Everson S, Frederick P, Eggers P, Agodoa L: Excerpts from the United States Renal Data System 2004 annual data report: Atlas of end stage renal disease in the United States Am J Kidney Dis 2005; 45(Suppl 1):A1
7. Rabbat CG, Thorpe KE, Russell JD, et al: Comparison of mortality risk for dialysis patients and cadaveric first renal transplant recipients in Ontário, Canadá. J Am Soc Nephrol 2000; 11: 917
8. West M, Sutherland DE, Matas AJ: Kidney transplant recipients who die with functioning grafts: serum creatinine level and cause of death. Transplantation 1996; 62: 1029
9. Lindholm A, Albrechtsen D, Frodin L, et al: Ischemic heart disease – major cause of death and graft loss after renal transplantation in Scandinavia. Transplantation 1995; 60: 451
10. Yeo FE, Villines TC, Bucci JR, et al: Cardiovascular risk in stage 4 and 5 hephrophaty. Adv Chronic Kidney Dis 2004; 11: 116
11. Braun J, Oldendorf M: EBCT in the evaluation of cardiac calcifications in chronic dialysis patients. Am J Kidney Dis 1996; 27: 394
12. Goodman WG, Goldin J, Kuizon BD, Yoon C, Gales B, Sider D, Wang Y, Chung J, Emerick A, Greaser L, Elashoff RM, Salusky IB: Coronary-artery calcification in young adults with end-stage renal disease who are undergoing dialysis. N Engl J Med 2000; 342: 1478
13. Russo D, [Palmiero G](http://www.ncbi.nlm.nih.gov/entrez/query.fcgi?db=pubmed&cmd=Search&itool=pubmed_AbstractPlus&term="Palmiero+G"%5BAuthor%5D), [De Blasio AP](http://www.ncbi.nlm.nih.gov/entrez/query.fcgi?db=pubmed&cmd=Search&itool=pubmed_AbstractPlus&term="De+Blasio+AP"%5BAuthor%5D), [Balletta MM](http://www.ncbi.nlm.nih.gov/entrez/query.fcgi?db=pubmed&cmd=Search&itool=pubmed_AbstractPlus&term="Balletta+MM"%5BAuthor%5D), [Andreucci VE](http://www.ncbi.nlm.nih.gov/entrez/query.fcgi?db=pubmed&cmd=Search&itool=pubmed_AbstractPlus&term="Andreucci+VE"%5BAuthor%5D): Coronary artery calcification in patients with CRF not undergoing dialysis. Am J Kidney Dis 2004; 44(6): 1024
14. Raggi P, Boulay A, Chasan-Taber S, Amin N, Dillon M, Burke S K, Chertow GM: Cardiac calcification in adult hemodialysis patients: a link between end-stage renal disease and cardiovascular disease? J Am Coll Cardiol 2002; 39 (4): 695
15. Matsuoka M, [Iseki K](http://www.ncbi.nlm.nih.gov/entrez/query.fcgi?db=pubmed&cmd=Search&itool=pubmed_AbstractPlus&term="Iseki+K"%5BAuthor%5D), [Tamashiro M](http://www.ncbi.nlm.nih.gov/entrez/query.fcgi?db=pubmed&cmd=Search&itool=pubmed_AbstractPlus&term="Tamashiro+M"%5BAuthor%5D), [Fujimoto N](http://www.ncbi.nlm.nih.gov/entrez/query.fcgi?db=pubmed&cmd=Search&itool=pubmed_AbstractPlus&term="Fujimoto+N"%5BAuthor%5D), [Higa N](http://www.ncbi.nlm.nih.gov/entrez/query.fcgi?db=pubmed&cmd=Search&itool=pubmed_AbstractPlus&term="Higa+N"%5BAuthor%5D), [Touma T](http://www.ncbi.nlm.nih.gov/entrez/query.fcgi?db=pubmed&cmd=Search&itool=pubmed_AbstractPlus&term="Touma+T"%5BAuthor%5D), [Takishita S](http://www.ncbi.nlm.nih.gov/entrez/query.fcgi?db=pubmed&cmd=Search&itool=pubmed_AbstractPlus&term="Takishita+S"%5BAuthor%5D): Impact of high coronary artery calcification score (CACS) on survival in patients on chronic hemodialysis. Clin Exp Nephrol 2004; 8(1): 54
16. Ammirati AL, Dalboni MA, Cendoroglo Neto M, Draibe SA, Canziani MEF: Coronary artery calcification, systemic inflammation markers and mineral metabolism in a peritoneal dialysis population”. Nephron Clin Pract 2006; 104 (1): 33
17. Barreto DV, Barreto FC, Carvalho AB, Cuppari L, Cendoroglo Neto M, Draibe SA, Moyses RMA, Neves KR, Jorgetti V, Blair A, Guiberteau R, Canziani MEF: Coronary calcification in hemodialysis patients: the contribution of traditional and uremia-related risk factors. Kidney Int 2005; 67: 1576
18. Tomiyama C, Higa A, Dalboni MA, Cendoroglo Neto M, Draibe SA, Cuppari L, Carvalho AB, Neto EM, Canziani ME: The impact of traditional and non-traditional risk factors on coronary calcification in pre-dialysis patients. Nephrol Dial Transplant 2006; 21: 2467
19. Moe SM, O’Niel KD, Resterova M, Fineberg N, Persohn S, Meyer CA: Natural history of vascular calcification in dialysis and transplant patients. Nephrol Dial Transplant 2004; 19: 2387
20. Oschatz E, Benesch T, Kodras K, Hoffmann U, Haas M: Changes of coronary calcification after kidney transplantation. Am J Kidney Dis 2006; 48: 307
21. Abbott KC, Bucci JR, Cruess D, et al: Graft loss and acute coronary syndromes after renal transplantation in the United States. J Am Soc Nephrol 2002; 13: 2560
22. Duclox D, Kazory A, CHalopin JM. Predicting coronary heart disease in renal transplant recipients: A prospective study. Kidney Int 2004; 66: 441
23. Humar A, Gillingham K, Payne WD, Sutherland DER, Matas AJ: Increased incidence of cardiac complications in kidney transplant recipients with cytomegalovirus disease. Transplantation 2000; 70(2): 310
24. Sarnak MJ, Levey AS. Cardiovascular disease and chronic renal disease: a new paradigm. Am J Kidney Dis 2000; 35 (Suppl. 1): S117
25. Arnadottir M, Hultberg B, Wahlberg J, et al: Serum total homocysteine concentration before and after renal transplantation. Kidney Int 1998; 54: 1380
26. Mix TCH, Pereira JGP, Kausz AT, et al: Anemia: A continuing problem following kidney transplantation. Am J Transplant 2003; 3: 1426
27. Romão JE Júnior, Haiashi AR, Elias RM, Luders C, Ferraboli R, Castro MC, Abensur H: Positive acute-phase inflammatory markers in different stages of chronic kidney disease. Am J Nephrol 2006; 26(1): 59
28. Stenvinkel P, Ketteler M, Johnson RJ, et al. IL-10, IL-6, and TNF-alpha: central factors in the altered cytokine network of uremia – the good, the bad, and the ugly. Kidney Int2005; 67:1216
29. Simmons EM, Langone A, Sezer MT, Vella JP, Recupero P, Morow JD, Ikizler TA, Himmelfarb J: Effect of renal transplantation on biomarkers of inflammation and oxidative stress in end-stage renal disease patients. Transplantation 2005; 79(8): 914
30. Cueto-Manzano AM, Morales-Buenrostro LE, Gonzáles-Espinosa L, Gonzáles-Tabaleros N, Martín-del-Campo F, Correa-Rotter R, Valera I, Alberú J: Markers of inflammation before and after renal transplantation. Transplantation 2005; 80 (1): 47
31. Ridker PM, Cushman M, Stampfer MJ, Tracy RP, Hennekens CH: Inflammation, aspirin, and the risk of cardiovascular disease in apparently healthy men. N Engl J Med 1997; 336: 973
32. Bakri RS, Afzali B, Covic A *et al*: Cardiovascular disease in renal allograft recipients is associated with elevated sialic acid or markers of inflammation. Clin Transplant 2004; 18: 201
33. Ross R: Atherosclerosis — An inflammatory disease. N Engl J Med 1999; 340(2): 115
34. Kocak H, Ceken K, Dinckan A, Mahsereci E, Yavuz A, Yucetin L, Akbas S, Gurkan A, Erdogan O, Ersoy F: Assessment and comparison of endothelial function between dialysis and kidney transplant patients. Transplantation Proc. 2006; 38(2): 416
35. Davignon J,Ganz P: Role of Endothelial Dysfunction in Atherosclerosis. Circulation 2004; 109: III-27
36. Steinberg D, Witztum JL. Is the oxidative modification hypothesis relevant to human atherosclerosis? Circulation 2002; 105: 2107
37. Gonyea JE, Anderson CF: Weight change and serum lipoproteins in recipients of renal allografts. Mayo Clin Proc 1992; 67: 653
38. Moore R, Thomas D, Morgan E, et al: Abnormal lipid and lipoproteins profiles following renal transplantation: Tranplant proc 1993; 25: 1060
39. Vanrenterghem Y, et al: Double-blind comparison of two corticosteroid regimens plus mycophenolate mofetil and cyclosporine for prevention of acute renal allograft rejection. Transplantation 2000; 70: 1352
40. Artz MA, et al: Improved cardiovascular risk profile and renal function in renal transplant patients after conversion from cyclosporine to tacrolimus. J Am Soc Nephrol 2003; 14: 1880
41. Kramer BK, Montagnino G, Del Castillo D, et al: Efficacy and safety of tacrolimus compared with cyclosporin A microemulsion in renal transplantation: 2 year follow-up results. Nephrol Dial Transplant 2005; 20: 968
42. Heaf J, Tvedegaard E, Kanstrup IL, Fogh-Andersen N: Hyperparathyroidism and long-term bone loss after renal transplantation. Clin Transplant 2003; 17: 268
43. Bellorin-Font E, Rojas E, Carlini RG, Suniaga O, Weisinger JR: Bone remodeling after renal transplantation. Kidney Int 2003; 85 (suppl): S125
44. Julian BA, Laskow DA, Dubovsky J, Dubovsky EV, Curtis JJ, Quarles LD: Rapid loss of vertebral mineral density after renal transplantation. N Engl J Med 1991; 325: 544
45. Monier-Faugere MC, Mawad H, Qi Q, Friedler RM, Malluche HH: High prevalence of low bone turnover and occurrence of osteomalacia after kidney transplantation. J Am Soc Nephrol 2000; 11: 1093
46. Almond MK, Kwan JT, Evans K, Cunningham J: Loss of regional bone mineral density in the first 12 months following renal transplantation. Nephron 1994; 66: 52
47. Epstein S: Post-transplantation bone disease: The role of immunosuppressive agents and the skeleton. J Bone Miner Res 1996; 11:1
48. **Baum C:** Weight gain and cardiovascular risk after organ transplantation. Journal of Parenteral and Enteral Nutrition. 2001; 25(3): 114
49. El-Agroudy AE, Wafa EW, Gheith OE, Shehab El-Dein AB, Ghoneim MA: Weight gain after renal transplantation is a risk factor for patient and graft outcome. Transplantation. 2004; 77(9):1381
50. Despres JP: Intra-abdominal obesity: an untreated risk factor for Typ2 diabetes and cardiovascular disease. J Endocrinol Invest 2006; 29(3 Suppl): 77
51. [Lau DC, Dhillon B, Yan H, Szmitko PE, Verma S](http://www.ncbi.nlm.nih.gov/entrez/query.fcgi?db=pubmed&cmd=Retrieve&dopt=AbstractPlus&list_uids=15653761&query_hl=9&itool=pubmed_docsum): Adipokines: molecular links between obesity and atheroslcerosis. Am J Physiol Heart Circ Physiol. 2005; 288(5):H2031
52. Seliger SL, Weiss NS, Gillen DL, Kestenbaum B, Ball A, Sherrard DJ, Stehman-Breen CO: HMG-CoA reductase inhibitors are associated with reduced mortality in ESRD patients. Kidney Int. 2002; 61(1):297
53. Massy ZA, Guijarro C: Statins: effects beyond cholesterol lowering Nephrol. Dial. Transplant. 2001; 16(9): 1738
54. [Wanner C, Krane V, Marz W, Olschewski M, Mann JF, Ruf G, Ritz E: German Diabetes and Dialysis Study Investigators.](http://www.ncbi.nlm.nih.gov/entrez/query.fcgi?db=pubmed&cmd=Retrieve&dopt=AbstractPlus&list_uids=16034009&query_hl=5&itool=pubmed_docsum) Atorvastatin in patients with type 2 diabetes mellitus undergoing hemodialysis. N Engl J Med 2005; 353(3):238
55. Åsberg A, Hartmann A, Fjeldså E, Holdaas H: Atorvastatin improves endothelial function in renal-transplant recipients. Nephrol Dial Transplant 2001; 16: 1920
56. Holdaas H, Fellström B, Cole E, Nyberg G, Olsson AG, Pedersen TR, Madsen S, Grönhagen-Riska C, Neumayer H-H, Mães B, Ambühl P, Hartmann A, Staffler B, Jardine AG: Long-term Cardiac Outcomes in Renal Transplant Recipients Receiving Fluvastatin: The ALERT Extension Study. Am J Transplant 2005; 5(12): 2929
